# Gamma Oscillations in the Temporal Pole Reflect the Contribution of Approach and Avoidance Motivational Systems to the Processing of Fear and Anger Words

**DOI:** 10.3389/fpsyg.2021.802290

**Published:** 2022-01-24

**Authors:** Gerardo Santaniello, Pilar Ferré, Alberto Sanchez-Carmona, Daniel Huete-Pérez, Jacobo Albert, José A. Hinojosa

**Affiliations:** ^1^Instituto Pluridisciplinar, Universidad Complutense de Madrid, Madrid, Spain; ^2^Universitat Rovira i Virgili, Department of Psychology, Research Center for Behavior Assessment (CRAMC), Tarragona, Spain; ^3^Facultad de Psicología, Universidad Autónoma de Madrid, Madrid, Spain; ^4^Facultad de Psicología, Universidad Complutense de Madrid, Madrid, Spain; ^5^Centro de Investigación Nebrija en Cognición (CINC), Universidad Nebrija, Madrid, Spain

**Keywords:** approach, avoidance, EEG, gamma band, beamforming, temporal pole

## Abstract

Prior reports suggest that affective effects in visual word processing cannot be fully explained by a dimensional perspective of emotions based on valence and arousal. In the current study, we focused on the contribution of approach and avoidance motivational systems that are related to different action components to the processing of emotional words. To this aim, we compared frontal alpha asymmetries and brain oscillations elicited by anger words associated with approach (fighting) motivational tendencies, and fear words that may trigger either avoidance (escaping), approach (fighting) or no (freezing) action tendencies. The participants’ task was to make decisions about approaching or distancing from the concepts represented by words. The results of cluster-based and beamforming analyses revealed increased gamma power band synchronization for fear words relative to anger words between 725 and 750 ms, with an estimated neural origin in the temporal pole. These findings were interpreted to reflect a conflict between different action tendencies underlying the representation of fear words in semantic and emotional memories, when trying to achieve task requirements. These results are in line with the predictions made by the *fear-hinders-action* hypothesis. Additionally, current data highlights the contribution of motivational features to the representation and processing of emotional words.

## Introduction

Language plays a pivotal role in communicating feelings and regulating social interactions. In the last few years, several event-related potentials and functional magnetic resonance imaging studies have investigated the neural underpinnings of emotional language, showing interactions between language and emotion at several processing stages during word, sentence and discourse comprehension (see Citron, 2012; Hinojosa et al., 2020, for reviews). Of note, research on the oscillatory neural activations associated with the processing of emotional words is very scarce, and has mainly relied on the assumptions of dimensional models of emotion (Russell, 2003). According to this view, valence (ranging from feeling unpleasant/negative to pleasant/positive) and arousal (ranging from feeling quiet to active) are the fundamental dimensions of affect. Thus, the emotional word *massage* refers to a positive and relaxing concept, whereas *shoot* denotes a negative and activating concept. In the study by Hirata et al. (2007), the authors observed a power decrease in the beta and gamma bands for both positive and negative words compared to neutral words that were associated with facilitated language processing during emotional word reading. Also, Wang and Bastiaansen (2014) reported an alpha power decrease for emotional words relative to neutral words that was interpreted in terms of attentional engagement during the processing of negative and positive high-arousing words.

Despite of the prevalence of dimensional models in behavioral and neurobiological research about the interplay between language and emotion, there is evidence indicating that approach and avoidance motivational directions (Davidson, 1993, 1995; Harmon-Jones et al., 2017) might also play a role in the processing of emotional words. In this sense, the valence-arousal conflict theory (Robinson et al., 2004) predicts that positive valence and low arousal are associated with approach-related action tendencies, while negative valence and high arousal are linked to avoidance behaviors. In line with this proposal, prior studies have shown that motivationally incongruent words (e.g., positive high arousing and negative low arousing words) are responded to more slowly than motivationally congruent ones (positive low arousing and negative high arousing words), although these effects were restricted to tasks that explicitly demanded approach-avoidance judgments from participants (Citron et al., 2014, 2016; Wang et al., 2018). Of note, in these studies the contribution of motivational directions to the processing of emotional words is subsidiary of the two affective dimensions of valence and arousal. To circumvent this limitation, a recent study compared the processing of fear and anger words that were matched in valence and arousal, but gave rise to different approach and avoidance motivational action tendencies (Huete-Pérez et al., 2019). In this sense, anger typically elicits approach-related behaviors (fight). In contrast, the dominant tendency evoked by fear is avoidance (flight), although this emotion may also prompt both approach (fight) or passive (freeze) action tendencies (Canon, 1929; Valk et al., 2015; LaBar, 2016). The results showed small size effects that consisted of delayed responses to fear words relative to anger words, which were again restricted to an approach-avoidance task (Exp. 3). The authors speculated that two possible explanations could account for their data. According to the *anger-fosters-action* hypothesis, approach motivational tendencies associated with anger would speed responses to these words relative to fear words. Alternatively, the *fear-hinders-action* hypothesis assumes that slower RTs to fear words reflect internal cognitive conflict and interference between avoidance (i.e., escaping), passive (i.e., freezing) and approach (i.e., fight) action tendencies, and/or the inhibition of incongruent motivational directions.

Together, evidence from these studies illustrate the need to consider motivational direction as separate from affective valence or arousal dimensions. However, neurobiological studies on the interplay between language and emotion have neglected the contribution of avoidance and approach action tendencies to the processing of affective language. To fill this gap, in the current study we analyzed brain oscillatory responses to anger and fear words matched in valence and arousal in an “approach-distancing” task to further test the predictions made by the *anger-fosters-action* and *fear-hinders-action* hypotheses regarding the processing of the motivational component of emotional words. To this aim, we assessed frontal alpha asymmetry (FAA), a difference score computed by subtracting the natural log of frontal left hemisphere alpha power from the natural log of frontal right hemisphere alpha power. Alpha band activity is inversely related to underlying cortical processing, since decreases in alpha power tend to be observed when underlying cortical systems engage in active processing. Therefore, higher FAA scores indicate relatively greater left frontal activity whereas lower scores suggest relatively greater right frontal activity (Coan and Allen, 2004; Briesemeister et al., 2013; Kelley et al., 2017). Of note, prior research has shown that FAA is a reliable correlate of motivational action directions, with increased left frontal activity indicating tendencies toward approach motivation (Davidson, 1993; Adolph et al., 2017; Harmon-Jones and Gable, 2018). Also, we analyzed oscillations in the beta-frequency and the gamma-frequency bands. Increased power in these bands have been proposed as a neural correlate of cognitive and response conflict, interference and inhibition (Sánchez-Carmona et al., 2019; Wiesman and Wilson, 2020; Wiesman et al., 2020; Schaum et al., 2021).

Predictions could be made as follows. If an *anger-fosters-action* mechanism drives motivational effects during the processing of fear and anger words, anger words associated with approach action tendencies should elicit higher right alpha activity (e.g., greater relative left vs. right frontal activation) relative to fear words. Alternatively, if prior motivational effects reflect conflict, interference and/or inhibition of incongruent action tendencies related to a *fear-hinders-action* mechanism, we would expect increased beta and/or gamma oscillatory power to fear words relative to angry words.

## Materials and Methods

### Participants

Our sample size was determined based on an *a priori* power analysis using G*Power (Faul et al., 2007). Assuming a α = 0.05 significance level, we estimated that a total sample size of 27 participants would provide 80% power to detect effects (medium size effect *d* = 0.5). Considering potential drop-outs, we recruited 33 Spanish native participants to exceed the criterion. Of the 33 recruited participants, 7 were excluded from further the analyses due to low overall task accuracy (out from 1.5 times the interquartile range). The remaining sample consisted of 20 females and 6 males aged 18–36 years (*M* = 20.42 years, *SD* = 3.45). All participants reported normal or corrected-to-normal vision and, with the exception of 3 left-handed participants, were right-handed according to the Edinburgh Handedness Inventory (Oldfield, 1971). They did not report any history of neurological disorders. Participants signed an informed consent before the experiment. The study was approved by the ethics committee at Instituto Pluridisciplinar.

### Stimuli

There were 35 anger words and 35 fear words. Since prior findings have shown that a “distancing” response should be expected for both fear and anger words (Huete-Pérez et al., 2019), we also selected 70 positive happiness-related words as fillers to match the number of “approach” responses in the task. Words were selected from several normative studies (Ferré et al., 2012, 2017; Guasch et al., 2016; Hinojosa et al., 2016a; Stadthagen-Gonzalez et al., 2017; Stadthagen-González et al., 2018) using the EmoFinder (Fraga et al., 2018). Both dimensional (valence, from *negative* to *positive*, and arousal, from *calmed* to *activated*, both in 9-points scale), and discrete (fear, anger, disgust, sadness and happiness, from *nothing at all* to *extremely*, all in a 5-points scale) affective ratings were considered. Fear and anger words had valences ratings < 4, and arousal scores ≥ 5. Fear words scored ≥ 3 in fear and ≤ 2.8 in anger, sadness, disgust and happiness. Similarly, anger words scored ≥ 3 in anger and ≤ 2.8 in other discrete emotions. Independent *t*-tests showed that fear words and anger words were matched in valence (*p* = 0.587), arousal (*p* = 0.129), happiness (*p* = 0.956), sadness (*p* = 0.455), disgust (*p* = 0.106), the target emotion (i.e., the average fear score of fear words vs. the average anger score for anger words; *p* = 0.129), and the contrast emotion (i.e., the average anger value for fear words vs. the average fear value for anger words; *p* = 0.305). Also, as illustrated in Table 1, stimuli were statistically matched (all *p* ≥ 0.096) in age of acquisition (Alonso et al., 2015; Huete-Pérez et al., 2019), concreteness and familiarity (Ferré et al., 2012; Duchon et al., 2013; Guasch et al., 2016; Hinojosa et al., 2016b; Huete-Pérez et al., 2019), number of higher frequency lexical neighbors, number of lexical neighbors, logarithm of contextual diversity, logarithm of lemma frequency, logarithm of word frequency, mean Levenshtein distance of the 20 closets words, number of syllables, and word length (Duchon et al., 2013). We used the K-means clustering procedure for this matching (Guasch et al., 2017). To avoid effects of grammatical category (Palazova et al., 2011), the number of nouns and words that could be considered both nouns and adjectives (Diccionario de la Lengua Española, RAE, 2014)^1^ was similar across conditions (fear words: 31 nouns and 4 nouns-adjectives; anger words: 30 nouns and 5 noun-adjectives). Finally, positive (filler) words were matched to both fear and anger words in these affective, sublexical, lexical and semantic variables with the exception of valence, discrete emotions, as well as the logarithm of lemma frequency, word frequency, and contextual diversity.

**TABLE 1:** Lexical, semantic and affective features of the experimental stimuli and the filler stimuli (standard deviations in parentheses).

	Fear words	Anger words	Positive-happiness words
Valence	3.07 (0.54)	3.01 (0.45)	7.06 (0.58)
Arousal	6.89 (0.56)	6.69 (0.54)	6.67 (0.61)
Happiness	1.29 (0.28)	1.29 (0.23)	3.71 (0.53)
Sadness	2.26 (0.32)	2.32 (0.35)	1.27 (0.20)
Fear	3.43 (0.38)	2.34 (0.33)	1.53 (0.40)
Anger	2.24 (0.48)	3.30 (0.30)	1.31 (0.23)
Disgust	1.96 (0.49)	2.15 (0.45)	1.26 (0.21)
Concreteness	4.89 (1.01)	4.55 (0.76)	4.67 (0.88)
Familiarity	5.07 (0.81)	5.21 (0.89)	5.34 (0.81)
Age of acquisition	7.72 (1.62)	7.70 (1.94)	7.77 (1.69)
Logarithm of word frequency	0.86 (0.54)	0.64 (0.56)	0.94 (0.49)
Logarithm of lemma frequency	3.51 (0.77)	3.18 (0.93)	3.68 (0.61)
Number of letters	8.00 (2.31)	8.17 (2.50)	7.74 (2.49)
Number of syllables	3.40 (0.95)	3.31 (0.80)	3.16 (0.96)
Number of lexical neighbors	2.80 (4.91)	3.14 (4.77)	3.00 (5.83)
Number of HF lexical neighbors	0.40 (1.44)	0.63 (1.59)	0.31 (0.81)
OLD20	2.27 (0.79)	2.25 (0.80)	2.18 (0.64)
Logarithm of contextual diversity	0.57 (0.42)	0.45 (0.42)	0.65 (0.38)

*The value indicated is the mean of all the words in that condition, and the standard deviations are in parentheses.*

*HF, higher frequency; OLD20, mean Levenshtein distance of the 20 closest words.*

### Procedure

The whole set of 140 words were randomly presented to each participants in a single block. A 10 trials practice block was allowed before the beginning of the experimental block. Each trial began with a fixation cross with a random duration from 500 to 1,000 ms. Thereafter, a word was presented until participants’ response or after a time limit of 3,500 ms. Participants performed an “approaching-distancing” task (Huete-Pérez et al., 2019). They were asked to think about the word’s referent and decide whether they would approach (e.g., *premio*/prize), or distance (e.g., *dinamita*/dynamite, *combate/*combat) themselves from it by pressing one of two different buttons (response buttons were counterbalanced). Participants performed the experimental task seated comfortably in an electrically shielded and sound-attenuated room. Task stimuli were presented on a computer monitor that was positioned at eye level about 65 cm in front of the participant. The task was designed and implemented in MATLAB, using Psychtoolbox.^2^

### EEG Recording

EEG activity was recorded from 62 Ag/AgCl electrodes mounted in an electrode cap (Electro-Cap International), arranged according to the International 10–10 System (American Electroencephalographic Society, 1991). All electrodes were referenced to the average of mastoids and their impedances were kept below 10 KΩ. In addition, the electrooculographic activity was recorded using vertical and horizontal bipolar electrodes. These electrodes were placed at supra-infraorbital level of the left eye and on the outer canthus of both eyes, respectively. Recordings were amplified using BrainAmp amplifiers (BrainProducts, Munich, Germany), continuously digitized at a sample rate of 1,000 Hz, and filtered online with a frequency band-pass of 0.01–100 Hz.

### Data Analysis

All statistical analyses involved a single factor with two levels (fear words, anger words). To test evidence against the null hypothesis, we conducted Bayesian analyses whenever the results from paired-samples *t*-test showed null findings. Positive words were not analyzed as they were filler stimuli.

#### Behavioral Analysis

We first removed the responses out of 2 standard deviations (SD) from the mean values of correct trials for each subject and condition. Thereafter, reactions times (RTs) outside the time range from + 300 to + 3,500 ms were also discarded. Both response speed and accuracy were analyzed with a paired-samples *t*-test analysis comparing fear words and anger words.

#### Time-Frequency Analysis

EEG data were analyzed with the Fieldtrip software package (Oostenveld et al., 2011),^3^ a toolbox implemented in the MATLAB environment (The MathWorks, Natick, MA). Only correct trials were included in the analysis. First, the continuous sets of raw data were re-referenced to the averaged mastoids and segmented into −1500 to 2000 ms epochs. Subsequently, an independent components analysis (Makeig et al., 1997) was performed to eliminate the blink artifacts (Jung et al., 2000). Finally, epochs with artifacts were individually rejected with a visual inspection criterion. Following this procedure, we retained, on average 29.58 (*SD* = 2.53) trials to anger words and 29.54 (*SD* = 2.42) trials to fear words. Time-frequency data were computed by convolving single trial data with a complex Morlet wavelet w (t, fo) having a Gaussian shape in time (δt) and a frequency (δf) around the center frequency (fo). This transformation allows an easy adaptation to balance the trade-off between temporal and frequency precision as function of frequency and produces smooth time-frequency plots easy to interpret (Cohen, 2014). Overlapping wavelets were centered at all frequencies comprised between 2 and 80 Hz, linearly spaced by two Hz steps. In order to adjust the balance between temporal and frequency precision as a function of frequency, the width of the wavelet increased from 3 to 7 cycles from low to high frequencies (Cohen, 2014). Finally, to normalize the resulted power, a decibel transformation was taken relative to baseline, defined from −500 to −300 ms before emotional words (dB_tf_ = 10log10[activity_tf_-mean(baseline_f_)]).

To test the *anger-fosters-action*, we calculated total frontal alpha power (8–13 Hz) for each participant and experimental condition before baseline normalization (e.g., Harmon-Jones, 2006). Thereafter, we normalized these distributions by log-transforming the power values for all electrodes. Finally, a FAA index was computed by subtracting the natural log of left alpha power from the natural log of the right alpha power. This measure was computed for F3/F4 electrodes comprising the whole epoch, starting from target stimuli onset. To statistically compare the relative frontal alpha activity between FFA indexes for fear and anger words, we conducted a paired-samples *t*-test.

To test the *fear-hinders-action* hypothesis examined the full spectrum of neural oscillations elicited by anger and fear words: theta (4–7.5 Hz), alpha (8–13 Hz), beta (14–30 Hz) and gamma bands (30–50 Hz). In each of these frequency bands, we followed a non-parametric randomization test with a clusters analysis approach (Maris and Oostenveld, 2007). This procedure controls for Type I error rate over electrodes and time. The spatial threshold to determine significant clusters was set at 2 channels. Differences between anger and fear words were explored with a parametric *t*-test, conducted for each time and electrode point. Spatio-temporal clusters were consequently identified as contiguous time points and electrodes groups with a *p*-value below.05. Cluster *p*–values were summed to obtain a cluster level test statistic. Only the cluster with the maximum statistical-level was considered. The significance of the test statistic was assessed by constructing a reference distribution of the cluster statistic. A cluster statistic histogram was obtained by calculating the cluster test statistic after randomly reassigning the data to each condition. After repeating this step over 1,000 times, *p*-values were then computed as the proportion of permutations that resulted in a larger observed cluster level statistic. Statistical analyses were performed for each frequency band. All permutation statistics were done using Fieldtrip.

#### Source Reconstruction

To estimate the neural origin of significant effects at the surface level, we followed a time domain linearly constrained minimum variance (LCMV) beamformer approach (Van Veen et al., 1997; Gross et al., 2001). This method tests for the likelihood of activity in every brain location using an optimized spatial filter that allows the maximization of the activity at the location of interest and the suppression of the external interfering activity. First, we computed a forward model to enhance the source specificity, based on a standardized realistic head. The volume conductor was distributed in a regular 3-D grid of 12 mm, and the leadfield matrix was calculated individually for each voxel. Subsequently, we computed the inverse model to obtain a spatial filter. Time segments were concatenated and re-referenced to the common average. Thereafter, the covariance matrix was calculated. Following this procedure, we obtained a common spatial filter. This filter was multiplied for the data of each experimental condition to estimate the source strength at grid points. Finally, data from the time-frequency decompositions were bandpass filtered around the target frequency band and the absolute value of the Hilbert transform computed from −500 to 800 ms for each condition and subject. To control against the power bias toward the center of the head, a baseline transform was performed before submitting source estimations to statistical analysis. At each grid location and for each subject and experimental condition, absolute power changes relative to baseline was calculated (post-stimulus power—pre-stimulus power).

Oscillatory power projections into the cortical source space for anger and fear-related words were compared using the non-parametric cluster-based permutations approach described above. Since time-windows were already defined by the results of time-frequency analyses, clusters were created relying only in the spatial dimension.

## Results

### Behavioral Results

On average 4% of trials were outliers. Similar RTs we found for fear words (*M* = 891 ms; *SD* = 0.152) and anger words (*M* = 889 ms; *SD* = 0.163) [*t*(25) = 0.341, *p* = 0.736, *d* = 0.018, BF_01_ = 4.574)]. Also, fear (*M* = 2.692, *SD* = 1.619) and anger words (*M* = 3.039, *SD* = 1.949) did not differ in accuracy [*t*(25) = −0.768, *p* = 0.449, *d* = 0.043, BF_01_ = 3.689).

### Time-Frequency Results

**FIGURE 1:**
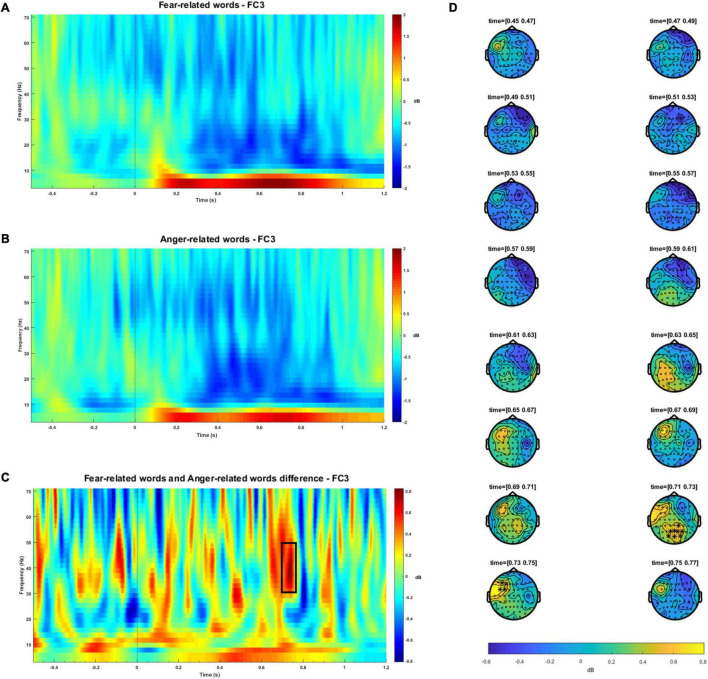
Time-frequency plots for the fear-related words **(A)** and anger-related words conditions **(B)**, for 4–70 Hz at a representative electrode location (FC3). To avoid artifact contamination, a -300 to -500 baseline prior stimulus target onset was used. Total power is expressed as decibel transformation relative to baseline. The black vertical line indicates the stimulus onset. **(C)** Time-frequency plot for the difference between fear-related words and anger-related words at a representative electrode (FC3). The black box highlights both the frequencies and the time range in which significant results were observed. The black vertical line indicates the stimulus onset. **(D)** Topographic distribution along the time course of the significant clusters observed in the gamma band (30–50 Hz) between fear-related words and anger-related words. Significant electrodes (*p* < 0.05) are highlighted with a black star. Color bar represents power difference between conditions, measured in decibels.

No significant differences were observed between fear and anger FAA indexes [*t*(25) = −0.614, *p* = 0.545, *d* = 0.119, BF_01_ = 4.062]. The results of time frequency analysis revealed increased activity in gamma power (cluster-based permutation test, *p* < 0.05) during the processing of fear words relative to anger words. These differences were observed at left fronto-central locations between 714 and 753 ms. A second significant effect was observed at right parieto-occipital sensors, starting from 690 up to 740 ms.^4^ These findings are illustrated in Figure 1. No differences were observed in theta, alpha or beta bands. Table 2 shows the results of the statistical analyses.

**TABLE 2:** *P*-values for the clusters in each frequency band analyzed.

Frequency-band	Cluster-based permutation test
Theta (4–7.5 Hz)	*p* = 0.0889 (positive cluster)
Alpha (8–13 Hz)	Unobserved positive/negative clusters
Beta (14–30 Hz)	*p* = 0.3337 (negative cluster)
Gamma (30–50 Hz)	*p* = 0.042 (positive cluster)

### Source Localization Results

**FIGURE 2:**
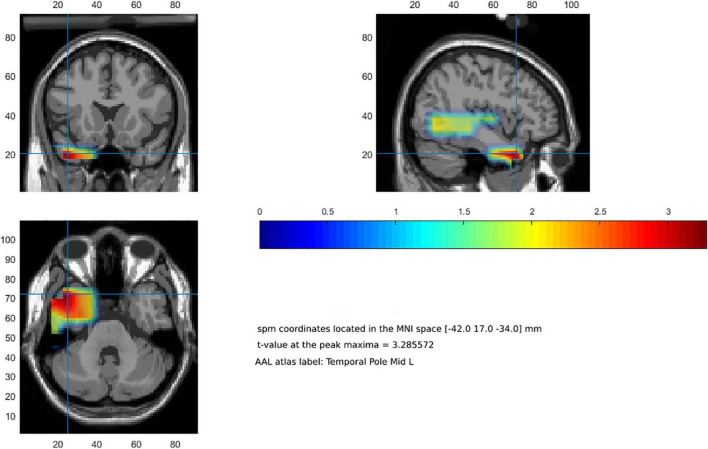
Beamforming reconstruction of the neural sources of gamma activity observed at the scalp level (fear-related words > anger-related words). Color bar represents *t*-values.

Beamforming analysis to estimate the neural origin of gamma band effects for fear words relative to anger words in the significant clusters yielded a peak maximum in the left temporal pole (BA 38; MNI coordinates x = −42, y = 17, z = −34). Figure 2 illustrates significant clusters (*p* < 0.05) from cluster-based permutation test.

## Discussion

In this study we further investigated the contribution of approach and avoidance motivational directions to the processing of emotional words. To this aim, we compared FFA and brain oscillations elicited by words denoting concepts associated with approach responses (i.e., anger words) with those evoked by words with conceptual referents related to conflicting action tendencies such as escape, fight of freeze (i.e., fear words). In line with prior reports, our data suggest a contribution of motivational systems to the processing of emotion words (Citron et al., 2014, 2016; Wang et al., 2018). As expected, participants gave distancing responses in the “approaching-distancing” task to both fear and anger negative words, whereas approaching responses were mainly restricted to positive words. Of note, RT differences between fear and anger words were not statistically significant, which contrasts with our prior findings (Huete-Pérez et al., 2019). This finding was unexpected since we only introduced slight changes in the current design (e.g., number of target stimuli). However, RT differences between fear and anger words in Huete-Pérez et al. study only emerged in the analyses by participants and they did not reach statistical significance in the analysis by items. All in all, these observations indicate that behavioral effects indexing the contribution of approach and avoidance systems to the processing of fear and anger words are rather weak. In contrast, our novel finding of increased gamma power to fear words compared to anger words suggests that brain activity might be a reliable index of the activation of approach and avoidance motivational systems in word processing.

Our study was designed to specifically test predictions made by two alternative explanations for prior results showing an influence of motivational systems in the processing of fear and anger words (Huete-Pérez et al., 2019). According to the *anger-fosters-action* view, a processing advantage for anger words could be expected in “approaching-distancing” tasks since these words are unequivocally associated with approaching, fight-related responses. In contrast, the *fear-hinders-action* hypothesis emphasizes the role of cognitive interference and the need to inhibit incongruent motivational directions associated with conflicting action tendencies underlying the representation of fear words, such as escaping, freezing or fighting.

The lack of FAA differences and the observation of increased activity in the gamma band for fear words relative to anger words favors an interpretation within the framework of the *fear-hinders-action* proposal since gamma oscillations have been related to conflict detection, interference and inhibition (Wiesman and Wilson, 2020; Wiesman et al., 2020), as well as the formation of memory for emotional experiences (Headley and Paré, 2013) amongst other functions. Interestingly, the results of our source analyses revealed that differences in gamma activity between fear words and anger words had an estimated neural origin in the left temporal lobe. This brain region is part of the associative limbic cortex or paralimbic cortex, and projects to other brain regions with a key role in emotional processing, such as the amygdala, the insula or the orbital prefrontal cortex (Chabardès et al., 2002; Olson et al., 2007; Herlin et al., 2021). The temporal pole has mnemonic functions related to the representation of conceptual knowledge in both semantic (Patterson et al., 2007; Ardila et al., 2014; Chadwick et al., 2016) and emotional (Dolan et al., 2000; Olson et al., 2007; Herlin et al., 2021) memories. Also, a critical role in binding highly processed linguistic and emotional information during the representation of semantic knowledge has been acknowledged (Olson et al., 2007). Thus, gamma activations in the temporal pole might reflect efforts to link different types of information about conflicting approaching, avoidance and freezing motivational action tendencies distributed in semantic and emotional memories underlying the conceptual representation of fear words. Of note, the timing of these EEG effects in relation to the RTs suggests that they seem to index the resolution of the task (i.e., competition between incompatible actions) rather than an automatic processing of word meanings.

To sum up, it has been widely established that the affective dimensions of valence and arousal influence the processing of emotion words (e.g., Kissler et al., 2007; Herbert et al., 2008; Méndez-Bértolo et al., 2011; Hinojosa et al., 2014). In contrast, with few exceptions, evidence regarding the contribution of approach and avoidance motivational systems to word processing is rather scarce (e.g., Citron et al., 2014, 2016; Huete-Pérez et al., 2019). Here we report a different pattern of brain activation for fear and anger words that were matched in arousal and valence, but were related to different motivational directions. Importantly, gamma band modulations in the temporal pole extend prior findings by showing that approach-withdrawn effects possibly arise from the conflict generated by the integration of difference sources of information about incongruent action tendencies involved in the conceptual representation of fear words, which is in line with the predictions made by the *fear-hinder-actions* hypothesis.

## Data Availability Statement

The raw data supporting the conclusions of this article will be made available by the authors, without undue reservation.

## Ethics Statement

The study was reviewed and approved by the Comité de Ética del Instituto Pluridisciplinar. The patients/participants provided their written informed consent to participate in this study.

## Author Contributions

GS, PF, DH-P, and JH contributed to the conception and design of the study. GS, AS-C, and JA performed the statistical analyses. GS and JH wrote the first draft of the manuscript. PF, AS-C, DH-P, and JA reviewed and edited the manuscript. All authors contributed to the article and approved the submitted version.

## Conflict of Interest

The authors declare that the research was conducted in the absence of any commercial or financial relationships that could be construed as a potential conflict of interest.

## Publisher’s Note

All claims expressed in this article are solely those of the authors and do not necessarily represent those of their affiliated organizations, or those of the publisher, the editors and the reviewers. Any product that may be evaluated in this article, or claim that may be made by its manufacturer, is not guaranteed or endorsed by the publisher.

## Footnotes

1
https://dle.rae.es

2
www.psychtoolbox.org

3
http://www.fieldtriptoolbox.org

4 There is some prior evidence showing handedness and gender effects in relation with approach-avoidance motivation and emotion (Brookshire and Casasanto, 2012; Kret and De Gelder, 2012; Hardie and Wright, 2014). To explore these potential interactions, we conducted additional analyses in the gamma band with handedness and sex. We failed to observe statistical differences in the gamma band between left-handed and right handed-participants (four non-significant negative clusters, all *p*s > 0.19), or between females and males (four non-statistically significant clusters, all *p*s > 0.59), for the processing of fear and anger words.
